# Rethinking social media metrics: Daily interaction valence and well‐being in university students

**DOI:** 10.1111/bjso.70109

**Published:** 2026-07-03

**Authors:** Aseel Sahib, Stephanie L. Hardacre, Olivia Evans, Anika S. Quayle, Mark Rubin, Alysia Robertson, Tegan Cruwys, Lillian Smyth

**Affiliations:** ^1^ School of Psychology, Deakin University Melbourne Australia; ^2^ School of Medicine and Psychology, the Australian National University Canberra Australia; ^3^ Centre of Excellence for Equity in Higher Education, the University of Newcastle Callaghan Australia; ^4^ Department of Psychology Durham University Durham UK

**Keywords:** daily diary, social connectedness, social media, social support, university well‐being

## Abstract

Rising concern about student mental health has highlighted the complex role of social media as both a source of support and a risk factor. However, existing research often relies on simplistic measures that overlook the context‐dependent ways in which students engage with these platforms. This daily diary study (159 observations; *N* = 40 university students) examined how the valence of university‐specific social media interactions related to student well‐being. Grounded in the view that digital spaces increasingly mediate campus social life, we tested whether more positive daily online interactions predicted greater subsequent well‐being and whether this association was mediated by sense of community and social support, provided or received. Social class was examined as a moderator. Multilevel analyses showed that more positive interaction valence was associated with higher subsequent daily well‐being, stronger sense of community, and greater provided and received social support. However, none of the mediators predicted well‐being, and no indirect effects were observed. These findings suggest that the quality of university‐related online interactions is linked to student well‐being.

University represents a critical period for well‐being, as students navigate major developmental and social transitions. Despite the potential for growth, many report lower well‐being and higher distress than their non‐student peers (Auerbach et al., 2018; Dingle et al., 2024; Rickwood et al., 2017; Crisp et al., 2025). Well‐being concerns are among the leading reasons students withdraw from university (Trusty et al., 2025), underscoring the importance of understanding how to support student well‐being.

Universities provide important opportunities for social engagement, a factor strongly associated with well‐being through its links to belonging, connection and perceived social support (Crawford et al., 2024; Pinto et al., 2024; Ruihua et al., 2025). However, as social interaction increasingly occurs online (Langehegermann & Samuel, 2025), many aspects of campus social life have similarly shifted to digital spaces. Students now frequently connect, collaborate and maintain relationships through online platforms and social media (Al‐Qaysi et al., 2023; Sutherland et al., 2021). While these environments can facilitate new forms of engagement, they may also reproduce or exacerbate existing social inequalities. For instance, students from working‐class backgrounds—who are more likely to combine study with paid employment—often have fewer opportunities to participate in university communities both offline and online, potentially limiting their sense of belonging and access to support (Rubin et al., 2019; Rubin & Wright, 2017).

## Social media use and mental health

Among university students, an estimated 85% report using social media for more than three hours daily (Osman, 2025). Given this high prevalence, considerable attention has been directed towards understanding whether social media use benefits or harms young people's well‐being. A substantial body of research has linked both general and problematic social media use to poorer mental health outcomes in young adults, with meta‐analytical evidence showing associations between social media use and higher psychological distress (Ahmed et al., 2024; Shannon et al., 2022). Proposed mechanisms include negative social comparisons (Sharma et al., 2020), sleep disturbances from night‐time usage (Alonzo et al., 2021) and fear of missing out (Roberts & David, 2020). Such findings have contributed to a dominant narrative that social media use is inherently detrimental to mental health, particularly among younger populations.

However, this conclusion is increasingly contested. Many studies in this literature are cross‐sectional, limiting causal inference (Valkenburg, 2022) and several studies suggest that individuals experiencing stress or low well‐being may increase their social media use as a coping response (Brailovskaia et al., 2019; Sun et al., 2023; Wolfers & Utz, 2022). At the same time, evidence for this explanation remains inconsistent. For example, using combined self‐report and objective measures, Griffioen et al. (2021) found no evidence that daily stress predicted subsequent social media use or that post‐stress use influenced momentary well‐being. These inconsistencies point to the need for methodologies capable of capturing temporal dynamics and within‐person processes, such as daily diary designs (Conte et al., 2025; Orben, 2020; Parry et al., 2022).

Beyond issues of causality, growing evidence suggests that the effects of social media appear to vary considerably across individuals. Ecological momentary assessment studies show substantial heterogeneity in affective responses following social media use, with some individuals reporting improvements, others a decline and many no change at all (Beyens et al., 2020). Such findings highlight that between‐person associations may obscure meaningful within‐person variation (Hall & Liu, 2022). Moreover, online experiences are shaped by broader contextual and structural factors (Evans & Rubin, 2022; Woodward et al., 2018), including socioeconomic background (Skogen et al., 2022; Visier‐Alfonso et al., 2024).

Another methodological challenge concerns how social media use is operationalized. Most studies rely on retrospective, time‐based estimates (e.g., hours of use), which correlate weakly with objective behaviour (Ellis et al., 2019; Ernala et al., 2020; Griffioen et al., 2020) and may obscure meaningful differences in the quality, purpose or content of online interactions (Flannery et al., 2023; Meier & Reinecke, 2021; Valkenburg, 2022). These limitations have prompted calls to move beyond quantity‐based metrics towards indicators that better capture the lived experience of social media use.

Consistent with this shift, a growing body of research highlights potential benefits of social media use, including associations with well‐being, life satisfaction, happiness and flourishing (Abebe et al., 2024; Janicke‐Bowles et al., 2023; Marciano & Viswanath, 2023; Ostic et al., 2021; Vaingankar et al., 2022). These positive outcomes are frequently attributed to social connection and support processes. Online interactions can foster belonging, maintain relationships and provide access to emotional or informational support that may be unavailable offline (Chen & Bello, 2017; Hu et al., 2017; Lo, 2019; Yue et al., 2024). Such findings align with social identity and ‘social cure’ frameworks, which posit that belonging to supportive social networks promotes psychological well‐being (Haslam et al., 2018; Jetten et al., 2012). Taken together, this work suggests that social media's potential benefits for well‐being are not inherent to platform use itself but emerge through the supportive qualities of everyday interactions. Understanding how social media affords well‐being therefore requires closer attention to the processes through which support is both given and received in online contexts.

### Giving and receiving social support online

Giving and receiving social support are central to psychological well‐being, shaping how individuals cope with stress, maintain resilience and build social bonds (Inagaki & Orehek, 2017; Ruihua et al., 2025). A substantial body of research demonstrates that receiving support can buffer stress and enhance mental health, while growing evidence suggests that providing support may offer unique and, in some cases, stronger benefits (Byrne et al., 2023). From a self‐determination theory (SDT) perspective, these benefits arise because supportive interactions satisfy fundamental psychological needs for relatedness (i.e., feeling connected to others) and competence (i.e., feeling effective and valued through one's actions; Ryan & Deci, 2000). When these needs are met, individuals are more likely to experience enhanced well‐being and sustained engagement in social relationships.

These processes also align closely with social identity and ‘social cure’ frameworks, which emphasize that belonging to meaningful social groups promotes health and well‐being by fostering shared identity, mutual support and a sense of collective value (Haslam et al., 2018; Jetten et al., 2012). Both giving and receiving support can strengthen social connectedness and reinforce an individual's identification with a broader community, which is an especially salient predictor of well‐being in university settings marked by academic and social transitions (Rubin et al., 2019).

These support processes are increasingly unfolding within digital spaces, where students turn to online platforms to seek advice, offer help and maintain social ties (Kluck et al., 2021; Zhou & Cheng, 2022). Research shows that online environments can facilitate meaningful support exchanges, with both giving and receiving support linked to increased well‐being, reduced loneliness and enhanced sense of community (e.g., Qian et al., 2023). Consistent with SDT and social identity perspectives, online interactions may be especially beneficial when they enable students to feel connected to others and to contribute in ways that affirm their value within a group. Importantly, these benefits do not appear confined to close relationships; even interactions within semi‐anonymous or loosely connected groups, such as university forums, course pages and student communities, can provide substantial emotional, informational and instrumental support (Sandstrom & Dunn, 2014). Taken together, this growing evidence highlights that online platforms are not merely social distractions but can serve as critical sites for sustaining students' relational worlds, offering daily opportunities to both draw on and contribute to social support networks.

## University‐specific social media

While general social media use has received widespread attention, a growing body of research highlights the unique potential of university‐specific social media platforms—such as course‐based Facebook groups, faculty‐managed discussion boards and peer‐run Discord servers—to enhance students' engagement, social support and sense of belonging (Ansari & Khan, 2020; Manca, 2020; Ngoc Hoi, 2023; Sabah, 2023; Zachos et al., 2018). These platforms function as digital extensions of the university environment, enabling students to interact, collaborate and exchange academic and social support beyond the confines of physical campus spaces.

Some of the most recent empirical evidence supports these benefits. Studies on Facebook‐based learning communities have found that students with access to official or peer‐led groups report a stronger sense of university belonging, closer peer relationships and greater course satisfaction compared to those without group access (Sheeran & Cummings, 2018; Thai et al., 2019). Similarly, in a randomized controlled trial, Zhou et al. (2023) demonstrated that participation in a peer‐led ‘study together’ Discord group increased classroom‐related belonging and improved academic performance—particularly among students with lower baseline motivation or academic preparation. However, the same intervention had less favourable outcomes for students who were already highly motivated, leading to poorer time management. Collectively, these findings indicate that while university‐specific social media can enhance connectedness and engagement, their effects are not uniform; they depend on individual characteristics such as motivation, learning preferences and pre‐existing engagement.

Despite these promising findings, a key gap remains. Existing reviews have summarized general links between social media use and mental health in university students (e.g., Ahmed et al., 2024) and between university‐specific platforms and engagement or belonging (e.g., Zachos et al., 2018), but no study to date has yet examined how university‐specific social media use influences student well‐being. Evidence from general social media research suggests that online interaction can foster social connection, belonging and perceived support – all of which are key pathways to well‐being (Oldenkamp, 2024; Taylor‐Jackson et al., 2021). It therefore remains to be tested whether university‐based platforms, which are uniquely embedded in students' academic and social ecosystems, can similarly promote well‐being by strengthening these support and connection mechanisms, and whether such benefits extend across different social contexts, including among students from varying socioeconomic backgrounds.

## The current study

Amid heightened concern about young people's social media use and its psychological consequences, empirically rigorous and context‐sensitive research is urgently needed. A growing body of reviews and meta‐analyses (e.g., Ahmed et al., 2024; Conte et al., 2025; Griffioen et al., 2020; Huang, 2022; Meier & Reinecke, 2021; Orben, 2020; Shannon et al., 2022; Valkenburg, 2022; Zhou & Cheng, 2022) have identified serious methodological limitations in this field – particularly the reliance on cross‐sectional designs and the simplistic operationalization of social media use as a time‐based metric. These limitations constrain understanding of how social media actually influences mental health and overlooks the contextual and individual factors that shape these effects (Parry et al., 2022). In response, scholars have increasingly called for designs that capture within‐person variability and day‐to‐day experiences, such as daily diary approaches, which reduce recall bias and can disentangle between‐ and within‐person processes (Bolger et al., 2003; Fisher & To, 2012; Janssens et al., 2018; Lischetzke et al., 2021; Miles, 2017; Silvia & Cotter, 2021).

This study focuses on social media use within a university‐specific context— namely, online interactions that occur within university environments (e.g., university groups, course‐specific group chats, faculty‐run pages). Unlike studies that quantify social media exposure in hours or frequency, it centres on interaction valence, the positivity or negativity of daily university‐specific online exchanges, as a more direct indicator of students' lived social experiences (Kuczynski et al., 2022; Norlin et al., 2025; Sun et al., 2020).

Social identity and social cure frameworks suggest that positive university‐based interactions may enhance well‐being by strengthening a sense of community and increasing opportunities to both provide and receive social support (Haslam et al., 2018; Jetten et al., 2012). These processes may not be uniform across students; however, as socioeconomic context shapes access to and benefits derived from social connection and support (Evans & Rubin, 2022; Woodward et al., 2018).

At the within‐person level, more positive (vs. negative) daily university‐specific interactions are expected to relate to higher well‐being, both directly and indirectly through sense of community, provided social support and received social support. In turn, greater community connection and supportive exchanges are expected to correspond with improvements in subsequent well‐being.

We hypothesize that on days when students report more positive university‐specific social media interactions, they will subsequently report: (H1a) greater sense of community, (H1b) higher provided social support and (H1c) higher received social support. Likewise, we hypothesize that more positive interaction valence (H2a), greater sense of community (H2b), higher provided (H2c) and received (H2d) social support would be associated with better subsequent well‐being. Furthermore, we hypothesis that the relationship between interaction valence and well‐being will be mediated by: (H3a) sense of community, (H3b) provided social support and/or (H3c) received social support. Finally, and more tentatively, we predict that the strength of these associations will be moderated by social class, such that effects of interaction valence on the mediators (e.g., sense of community) may vary by students' social class (H4a‐c). This was an exploratory investigation, so while we expected differences by social class, we did not have specific predictions about the direction of these effects.

## METHOD

### Participants and procedure

Data were collected across seven days in October 2023 within the academic teaching period, from Monday through to Sunday. Eligibility criteria included: enrolment at an Australian university since at least February 2023, an active social media account (i.e., an active account with any social media networking sites such as Facebook, Instagram, Twitter (X), TikTok, WhatsApp, Snapchat, Facebook Messenger, Telegram, Signal, LinkedIn, Reddit, Tumblr, BeReal, etc.) and being 18 years old or over. Recommended sample sizes for daily diary studies vary greatly—from as low as five to upwards of hundreds of participants (Lischetzke et al., 2021). To ensure sufficient power, factors such as design, the multilevel structure of the data, intended analyses, expected number of responses per participant, and lost data due to missed responses and attrition must be considered (Ohly et al., 2010). Attrition is typically higher than usual for daily diary studies, given the more intensive nature of participation (Ohly et al., 2010); therefore, to mitigate this, we aimed to recruit 55 participants. In total, 53 students signed up to participate, however 13 of these participants were excluded because they did not complete at least one of the seven daily surveys—a common minimum inclusion criterion for daily life research (Conner & Silvia, 2015). Our final sample consisted of 40 students from multiple Australian universities, including 28 participants recruited through Prolific and 12 participants recruited through snowball sampling. The total number of observations was a range between 163 and 214. Just over half of the participants were women, with a mean age of 28.57 years (SD = 10.40, median = 23.00; range: 18–60 years). Table 1 presents full demographic information.

**TABLE 1:** Participant demographics.

	*N* = 40
	%
Gender	32.1% Man
	58.5% Woman
	3.8% Non‐binary
Ethnicity	45.3% White
	3.8% Aboriginal and/or Torres Strait Islander
	47.5% Ethnic Minority
Age Group	37.5% Younger than 21 years
	55% Mature age student (21 years and over)
Student Type	75.5% Domestic
	18.9% International
Enrolment Type	71.7% Full‐time
	22.6% Part‐time
Program Type	67.9% Undergraduate
	26.5% Postgraduate
Year of University Study	17.0% First
	39.6% Second
	22.6% Third
	15.1% Fourth
Study Mode	75.5% On campus
	18.9% Distance/Online
First in Family Status	24.5% Yes
	69.8% No
Relocated for University	30.2% Yes
	64.2% No

*Note*: These percentages capture responses made. As there was some missing data, they do not always add up to 100%.

Participants completed brief online surveys using the secure browser‐based application REDCap. Participants were asked to regularly check their text messages during the study to ensure they did not miss survey prompts. The surveys were sent between the hours of 6 pm and 11 pm. Participants who had not completed a survey two hours after the initial prompt was sent received a reminder to participate. Participants completed the surveys online using their own device (e.g., phone, computer) to access the survey links. The ethical aspects of this research were approved by the Australian National University's Human Research Ethics Committee (protocol 2021/817).

### Measures

Because of the sampling frequency of the study, brief single‐item measures were employed where appropriate to reduce the burden on participants and to optimize compliance and data quality (Eisele et al., 2022). Where possible, validated measures were used, with the researchers devising any additional required measures after reviewing relevant literature. See Table 2 for the reliability results of the current study.

**TABLE 2:** Descriptive statistics.

	*N*	Mean	SD	ICC	Within ω [95% CI]	Between ω [95% CI]	1	2	3	4	5	6
(1) Valence	163	5.01	1.09	.29	‐	‐	‐					
(2) Sense of Community	204	4.64	1.39	.28	.92 [.89–.94]	.81 [.61–1.00]	.35*	‐				
(3) Provided Social Support	202	2.82	2.6	.31	.88 [.85–.91]	.85 [.56–1.00]	.28	.48**	‐			
(4) Received Social Support	202	2.64	2.53	.21	.88 [.84–.91]	.90 [.75–1.00]	.40*	.48**	.76**	‐		
(5) Well‐being	214	4.15	0.85	.63	.73 [.65–.81]	.92 [.86–.99]	.20	.16	.04	.03	‐	
(6) Social Class	40	4.96	1.20	‐	‐	‐	−.37*	−.16	.21	.10	−.20	‐

*Note*: As a single‐item variable, reliability could not be computed for interaction valence. The ω for social class was.90. * = *p* < .05; ** = *p* < .01.

Abbreviation: *N*, number of observations.

#### Interaction valence: Predictor variable (X)

**FIGURE 1:**
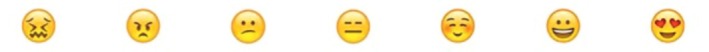
Anchors from K‐State Emoji Scale (Swaney‐Stueve et al., 2018). From left to right, the first three emojis represent negatively valenced emojis, then a neutral face, with the remaining three emojis capturing positively valenced emojis.

To assess the emotional valence of interactions (i.e., positive or negative valence) that participants had with other students on social media that day, we adapted Keil et al.'s (2020) approach using an emoji scale. Participants were asked, ‘How did your interactions with other students from your university on social media make you feel today?’, with response options based on the seven emoji anchors from Swaney‐Stueve et al.'s (2018) K‐State Emoji Scale (see Figure 1). An eighth option was also provided, for days when participants did not have a university‐specific social media interaction and were coded as missing: ‘Not applicable, I have not had social media interactions with fellow uni students in the last 24 hours’. Emoji response anchors were chosen because they offer a quick, intuitive method for participants to provide data (Deubler & Swaney‐Stueve, 2020).

#### Sense of community: Mediator (M)

Participants' daily social connectedness with fellow students from their university was measured using the 3‐item Sense of Community scale (Oh et al., 2014). They were asked: ‘Thinking about your university‐related activities/interactions on social media today, please indicate how true the following statements are for you’. The measure used a 7‐point rating scale ranging from 1 (d*efinitely false*) to 7 (d*efinitely true*) and included items such as ‘I felt a sense of contact with people who care for me’ and ‘I felt connected with others who are important to me’. Responses to the three items were averaged to compute a total sense of community score, with higher scores indicating greater perceived social connectedness.

#### Social support: Mediators (M)

Both provided and received social support were measured using items adapted from Oh et al. (2014). Participants were instructed as follows: ‘Thinking about your university‐related activities/interactions on social media, please indicate whether you have provided or received each of the following types of support since your last survey: (1) giving advice; (2) showing empathy; (3) validating thoughts; (4) complimenting; (5) teaching something new; (6) inviting to a new group; (7) sharing information; (8) giving encouragement; (9) inviting to a group plan; (10) Other (please specify)’. For each item, participants selected ‘yes’ or ‘no’ to indicate whether they had (a) provided and (b) received that type of support. The total number of supportive interactions was calculated by summing positive responses separately for provided and received support, resulting in scores ranging from 0 to 10 per person, for both types.

#### Well‐being – Outcome variable (Y)

Well‐being was measured using the five‐item Mental Health Inventory‐5 (Berwick et al., 1991), which assesses mental health and symptoms of depression and anxiety. Participants were asked, ‘How much of the time today: (1) Have you been a happy person? (2) Have you felt calm and peaceful? (3) Have you been a very nervous person (reversed)? (4) Have you felt downhearted and blue (reversed)? (5) Have you felt so down in the dumps that nothing could cheer you up (reversed)?’ Responses were recorded on a 6‐point scale ranging from 1 (*None of the tim*e) to 6 (*All of the time*), with higher scores indicating better well‐being.

#### Social class – Moderator variable

Social class was assessed using the Comprehensive Social Class Scale (Evans et al., 2022), which is designed for higher education populations. The scale comprises 11 items capturing aspects of parental, familial and personal background. These include both objective indicators, such as parental education, occupational prestige and family income during childhood, and subjective indicators, such as self‐identified social class and perceived social status. Following the procedure outlined by Evans et al. (2022), each item was converted to a *z*‐score and then aggregated to create a composite score, with higher scores indicating higher social class membership.

### Statistical analysis plan

As daily diary data are nested in multiple levels—day‐level observations within people—multilevel modelling procedures were used to parcel out within‐person variance from between‐person variance (Leyland & Groenewegen, 2020). This decision was further supported by our intraclass correlations (see Table 2), with a range of 21–63% of the variance being explained at the individual level (Peugh, 2010). There was a maximum of 280 observations (Level 1) nested in 40 people (Level 2).

As is common with daily diary studies, there was a moderate amount of missing data (21.6% for the mental health outcome variable; between 21.6% and 26.0% of data missing for the predictors). To handle this, we used multiple imputation via the mice package (van Buuren & Groothuis‐Oudshoorn, 2011), with imputation methods appropriate for our multilevel data structure. That is, we used the 21.pan method for the daily (Level 1 variables) and the 21.only.norm method for social class (Level 2). Participant ID was specified as the clustering variable in the predictor matrix. We generated 20 imputed data sets and then fit the multilevel mediation models to each imputed data set, pooling the parameter estimates across the imputations in line with Rubin (1987; i.e., using the pool() function in mice).

**FIGURE 2:**
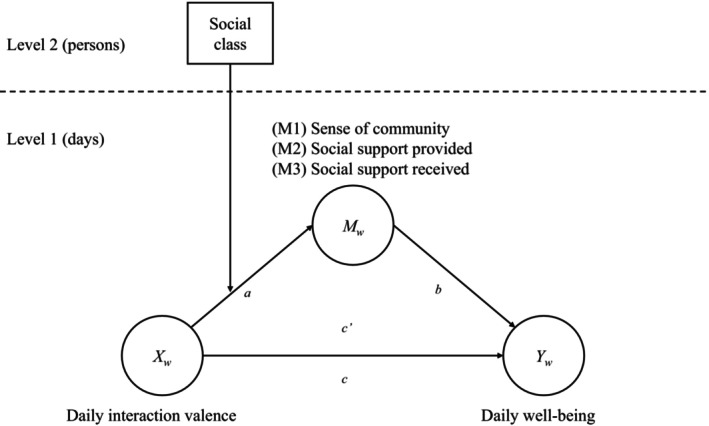
Moderated multilevel (1‐1‐1) mediation model. Moderated multilevel (1‐1‐1) mediation model (First‐Stage Multilevel Conditional Process Model). Index W indicates the within‐person part of the time‐varying variables.

Three moderated multilevel (1‐1‐1) mediation models were conducted using the reshape2, dplyr, tidyverse, lme4, lmerTest and boot packages in *R* (Version 2026.04.0 + 526; that is, a first‐stage multilevel conditional process model; cf. Hayes & Rockwood, 2020). These models tested whether the association between daily (evening) interaction valence (predictor X) and daily well‐being (outcome variable Y) was mediated by either (a) sense of community, (b) social support provided or (c) social support received (mediators M) and whether this indirect effect was moderated by participants' social class (cross‐level interaction; see Figure 2). Random intercepts were specified for participants to account for repeat observations for each person, and models were estimated using maximum likelihood.

Aligning with our hypotheses, we built these models by first testing whether valence predicted each mediator (sense of community, social support provided or social support received; H1a‐c) and whether this association was moderated by social class (path a; H4a‐c). If the interaction was non‐significant, we proceeded with a standard multilevel mediation model without moderation. Next, we tested whether valence predicted well‐being (H2a; total effect, path c) and whether each mediator predicted well‐being when controlling for valence (H2b‐d; path b). The direct effect of valence on well‐being (path c) was estimated to examine whether associations between valence and well‐being remained after accounting for each mediator. Lastly, indirect effects were estimated by bootstrapping (1000 resamples) within each imputed dataset and combining the resulting percentile‐based confidence intervals across imputations (H3a‐c). All daily/Level 1 variables were person‐centred (cluster‐mean centred). Social class was grand‐mean centred and entered as a Level 2 moderator. See Supplementary Materials for R code.

## RESULTS

### Preliminary results

Table 2 provides the means, standard deviations, intraclass correlations, McDonald's omega values at the within and between‐person level, and correlation matrix of person‐centered variables. Table 3 provides the details regarding the most and least selected type of social support. The most selected type of social support for both provided and received was ‘sharing information’, while the type of social support provided and received the least was ‘inviting to a new group’.

**TABLE 3:** Types of social support provided and received.

	Provided	Received
Sharing information	111	115
Validating thoughts	85	84
Giving encouragement	83	67
Showing empathy	82	78
Giving advice	81	76
Complimenting	71	55
Teaching something new	31	36
Invitation to a group plan	17	17
Invitation to a new group	9	8
Other (please specify)	0	0

*Note*: Scores are the total sum of ‘Yes’ responses selected by the 40 participants over the seven days (i.e., yes provided, yes received). The actual minimum and maximum sums for each type of social support were 0 and 115, respectively, out of a possible maximum of 280.

### Main results

#### Sense of community

In support of H1a, there was a significant within‐person effect of valence on sense of community, such that participants reported a greater sense of community on days when they experienced more positive social media interactions than usual, *b* = .43, SE = .15, *p* < .01. In contrast to H4a, the effect of valence on sense of community was not moderated by social class, *b* = .01, SE = .02, *p* = .620, and no main effect of social class emerged. As such, a standard mediation model was estimated.

Consistent with H2a, there was a significant total effect of daily interaction valence on daily well‐being (path c), indicating that participants reported higher well‐being on days when they experienced more positive interactions. When sense of community was included in the model, the direct effect of valence on well‐being (path c') remained significant, *b* = .09, SE = .05, *p* = .05. However, sense of community did not significantly predict well‐being when controlling for valence, *b* = .05, SE = .03, *p* = .168 (H2b).

**FIGURE 3:**
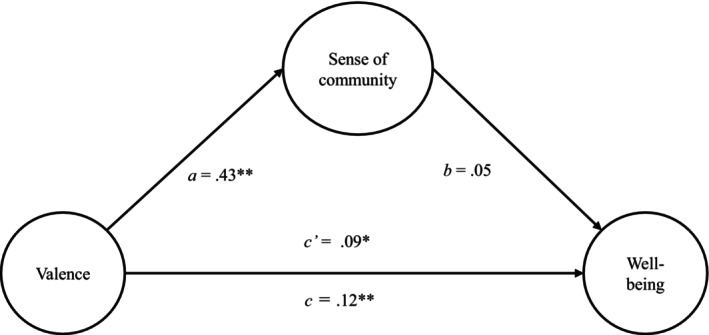
Mediation model examining the indirect effect of daily interaction valence on daily well‐being through sense of community. * = *p* < .05, ** = *p* < .01. The indirect effect was *b* = .02, 95% CI [−.002, .061].

The bootstrapped indirect effect of valence on well‐being via sense of community was not statistically significant, *b* = .02, 95% CI [−.002, .061]. Contrary to H3a, these findings indicate that although positive university‐specific social media interactions were associated with both greater sense of community and higher well‐being, sense of community did not mediate the association between interaction valence and well‐being (see Figure 3). Given that the confidence interval narrowly crossed zero and the indirect effect accounted for a meaningful proportion of the total effect (16.67%), it is possible that significance would have been reached with greater statistical power.

#### Social support provided

In support of H1b, there was a significant within‐person effect of valence on support provided, such that participants reported providing more support to others on days when they experienced more positive social media interactions than usual, *b* = .74, SE = .27, *p* < .01.^1^ In contrast to H4b, the effect of valence on support provision was not moderated by social class, *b* = −.01, SE = .04, *p* = .885, and no main effect of social class emerged. As such, a standard mediation model was estimated.

Consistent with H2a, there was a significant total effect of daily interaction valence on daily well‐being (path c). When social support provided was included in the model, the direct effect of valence on well‐being (path c') remained significant, *b* = .12, SE = .05, *p* = .022. However, social support provided did not significantly predict well‐being when controlling for valence, *b* = −.01, SE = .02, *p* = .674 (H2c).

**FIGURE 4:**
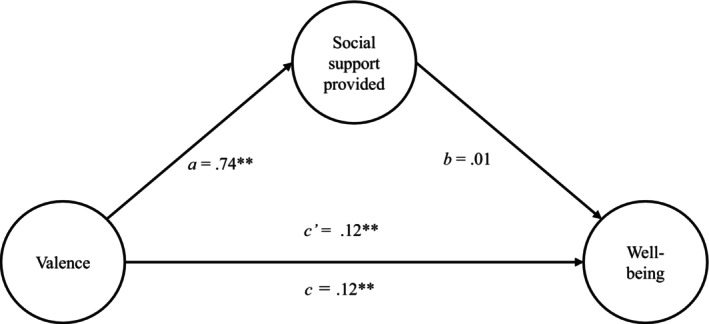
Mediation model examining the indirect effect of daily interaction valence on daily well‐being through social support provided. * = *p* < .05, ** = *p* < .01. The indirect effect was *b* = −.01, 95% CI [−.030, .013].

The bootstrapped indirect effect of valence on well‐being via social support provided was not statistically significant, *b* = −.01, 95% CI [−.030, .013]. Contrary to H3b, these findings indicate that although positive university‐specific social media interactions were associated with both greater support provision and higher well‐being, support provided did not mediate the association between interaction valence and well‐being (see Figure 4). The proportion of total effect accounted for by the indirect effect was small (8.33%), suggesting that the direct effect accounts for nearly all the total effect.

#### Social support received

In support of H1c, valence significantly predicted social support received, *b* = 1.03, SE = .25, *p* < .001, such that participants reported receiving more support on days when they experienced more positive social interactions than usual. As with support provided and in contrast to H4c, the effect of valence on support received was not moderated by social class, *b* = −.001, SE = .04, *p* = .988, and no main effect of social class emerged. A standard mediation model was therefore used.

Consistent with H2a, there was a significant total effect of daily interaction valence on daily well‐being (path c). When social support received was included in the model, the direct effect of valence on well‐being (path c') remained significant, *b* = .13, SE = .05, *p* = .021. However, social support received did not significantly predict well‐being when controlling for valence, *b* = −.002, SE = .02, *p* = .438 (H2d).

**FIGURE 5:**
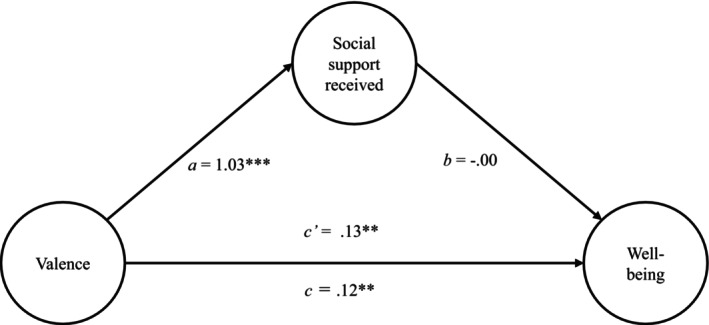
Mediation model examining the indirect effect of daily interaction valence on daily well‐being through social support received.* = *p* < .05, ** = *p* < .01, *** = *p* < .001. The indirect effect was *b* = −.02, 95% CI [−.047, .014].

The bootstrapped indirect effect of valence on well‐being via social support received was not statistically significant, *b* = −.02, 95% CI [−.047, .014]. Contrary to H3c, these findings indicate that although positive university‐specific social media interactions were associated with both greater received support and higher well‐being, received support did not mediate the association between interaction valence and well‐being (see Figure 5). Given that the confidence interval narrowly crossed zero and the indirect effect accounted for a meaningful proportion of the total effect (16.67%), it is possible that significance would have been reached with greater statistical power.

## DISCUSSION

The present study examined whether university‐specific social media interactions are linked to students' concurrent daily well‐being and whether this association operates through social connectedness (sense of community), provided social support or received social support. We also tested whether students' social class moderated the relationship between interaction valence and these potential mediators.

We found full support for Hypothesis 1. On days when students reported more positive university‐specific social media interactions, they also reported a greater sense of community (H1a), higher provided social support (H1b) and higher received social support (H1c). Hypothesis 2 was only partially supported: more positive interaction valence was associated with greater daily well‐being (H2a; as a direct and total effect), but sense of community, provided support, and received support were not directly associated with daily well‐being (H2b‐H2d). In line with these results, Hypothesis 3 was not supported—none of the proposed mediators explained the link between interaction valence and well‐being. Finally, Hypothesis 4 was also not supported: social class did not moderate the association between interaction valence and any of the mediators (H4a‐c).

### The context‐dependent benefits of social media use

This study shows that the quality of online interactions shapes student well‐being, though not through the pathways we anticipated. Aligned with prior empirical work (e.g., Abebe et al., 2024; Beyens et al., 2020; Janicke‐Bowles et al., 2023; Marciano & Viswanath, 2023; Ostic et al., 2021; Vaingankar et al., 2022), we found that more positively rated university‐specific interactions predicted greater social connectedness, social support (provided and received) and well‐being among university students. Drawing on SDT, recent reviews have highlighted that social media use can enhance well‐being when it fulfils core psychological needs (Ryan & Deci, 2000; West et al., 2024a). For instance, young people often use social media to seek belonging and acceptance, fulfilling the need for relatedness (e.g., Huang, 2021), while other studies show that sharing and receiving knowledge through social media relates to the need for competence (e.g., Asterhan & Bouton, 2017). Importantly, this research emphasized the *quality and purpose* of interactions (West et al., 2024b). Our study adopted a similar approach by focusing on interaction valence, instead of time‐based metrics of social media use. In line with SDT, we found that positive interaction experiences predicted outcomes related to relatedness (social connectedness), competence (support provided and received) and well‐being. Measuring perceptions of interactions in near real time may have captured whether students felt good as a result of these exchanges, helping to explain the consistent positive associations across outcomes.

Despite emerging and theoretically consistent associations between social media use and each proposed social mediator—sense of community, social support provided and social support received (Haslam et al., 2018; Jetten et al., 2012; Yue et al., 2024; Zhou & Cheng, 2022)—none of these variables explained the valence‐well‐being association. This pattern suggests that while positive interactions may foster social connectedness and support‐related behaviours, these social processes do not appear to be the primary mechanisms through which interaction valence translates into immediate fluctuations in well‐being. Instead, the benefits of positive social media interactions may operate through more proximal affective or cognitive pathways, such as momentary positive affect (Graciyal & Viswam, 2021) or feelings of validation (Onifade, 2022), which were not directly captured by the social mediators examined here. Alternatively, social support and community may exert more cumulative or longer‐term influences on well‐being that are not readily detectable at the daily timescale. Together, these findings highlight the importance of distinguishing between social correlates and social mechanisms in daily social media research and suggest that the well‐being benefits of positive online interactions may be more experiential than relational in the short term.

Finally, social class did not moderate the relationship between interaction valence and sense of community, nor either form of social support. While this finding may suggest a lack of meaningful social class differences in these processes, we believe that it is more plausible that the null effects observed here are attributable to methodological limitations (e.g., restricted variability in the sample, measurement constraints and/or insufficient statistical power). This interpretation is also supported by a substantial body of literature documenting that social class shapes students' experiences within university contexts, including their patterns of social interaction, access to support and feelings of belonging (Evans & Rubin, 2022; Woodward et al., 2018). As such, the present findings should not be taken as definitive evidence against the role of social class. Notably, this study represents one of the first to examine these dynamics in a university setting using this specific framework. Further research, employing more sensitive measures and diverse samples, is therefore necessary to more accurately assess whether and how social class may moderate these relationships.

### Implications

These findings have important implications for how social media use is studied, and our understanding of how social media is used by students in higher education. At a research level, our results directly address longstanding concerns about methodological limitations in social media research. Reviews and meta‐analyses have noted the overreliance on cross‐sectional designs and simplistic time‐based metrics (Conte et al., 2025; Meier & Reinecke, 2021; Shannon et al., 2022; Valkenburg, 2022), which restrict understanding of how social media influences mental health in specific contexts, such as universities (Parry et al., 2022). By focusing on university‐specific online interactions and their daily perceived valence, our study demonstrates that, when context is specified, social media use can be positively linked to students' connectedness, support and well‐being (cf. Ahmed et al., 2024). This is important as social media is here to stay and is used for increasingly longer times (~ 6.5 new users join social media every second and the overall time spent on social media has increased from 90 min in 2012 to 141 min in 2025; Kepios, 2025a, 2025b). Understanding how social media can actively support—rather than simply undermine— young people's well‐being is therefore a critical direction for future research, one that requires methods capable of capturing these processes as they unfold in daily life.

Our use of a daily diary design responds to growing calls for methods that capture day‐to‐day fluctuations, reduce recall bias and allow distinctions between within‐person and between‐person effects (Iida et al., 2023; Silvia & Cotter, 2021). These methodological advantages provide a clearer and more dynamic picture of how online interactions shape student well‐being, underscoring the importance of using such designs more widely in future research.

### Limitations and future directions

While our findings offer valuable insights, they should be interpreted in light of some methodological limitations that point to directions for future research. One key limitation concerns sample size. Although the study design enabled rich within‐person analyses, a larger sample would improve statistical power and allow for more precise estimation of the proposed models. This study was also not pre‐registered, which may limit transparency regarding the distinction between a priori hypotheses and analytic decisions made during the research process. However, the analyses were guided by established theoretical frameworks and existing empirical literature. Future studies would benefit from pre‐registration to strengthen inferential clarity and reduce potential researcher degrees of freedom, particularly in mediation analyses.

More broadly, there is scope to extend the methodological approach. Although the daily diary design captures meaningful day‐to‐day variation and reduces recall bias, future research could build on this work by employing more intensive longitudinal methods—such as ecological momentary assessment—to better capture interactions as they unfold in real time (Bolger et al., 2003; Fisher & To, 2012). Such approaches would provide an even more fine‐grained understanding of how online experiences relate to well‐being in situ.

A further limitation relates to the scope of inequality examined in this study. While we focused on social class as an important structural factor, university populations are diverse across a range of dimensions. Future research should therefore consider how other forms of diversity may shape the extent to which positive online interactions translate into social connectedness, support and well‐being (Gonzales, 2015; Parry et al., 2022).

At the same time, our measures of social connectedness and social support were relatively broad, capturing the presence of support or feelings of community rather than whether support met participants' needs or whether the recipients of support felt positively about it (Kuczynski et al., 2022; Norlin et al., 2025). Developing more nuanced, context‐sensitive measures would allow future work to better assess the quality and meaningfulness of these interactions. For example, adapting the short form of the Social Support Questionnaire—which includes a section for rating one's satisfaction with their social support—to be university‐specific (Sani et al., 2012; Sarason et al., 1987).

Addressing these limitations will not only strengthen the methodological rigour of future studies but also provide a deeper understanding of how social media can promote well‐being across diverse student populations and potentially reduce or reinforce inequities in online and offline experiences (Huang, 2022; Meier & Reinecke, 2021; Orben, 2020).

## CONCLUSION

This study provides new evidence about how university‐specific social media interactions relate to students' daily well‐being. By adopting a context‐sensitive and daily diary approach, we demonstrated that the valence of online interactions predicts social connectedness, social support and overall well‐being.

Beyond these empirical contributions, the findings also prompt a reconsideration of the mechanisms linking social media use to well‐being. The absence of mediation by context‐specific measures of connectedness and support suggests that commonly assumed social pathways may not operate in the short term as anticipated. This highlights the importance of distinguishing between social correlates and underlying mechanisms, and points to the need for theoretical models and measurement approaches that more precisely capture how and when social media experiences translate into well‐being.

Taken together, these findings advance theoretical understanding of social media's role in student mental health, and demonstrate the value of rigorous, context‐sensitive and within‐person designs. Future work that builds on these strengths—particularly through more intensive longitudinal methods and more diverse samples—will be critical to fully understanding how online interactions contribute to well‐being across student populations, and whether these processes mitigate or reinforce existing inequalities.

## AUTHOR CONTRIBUTIONS

**Aseel Sahib:** Writing – original draft; methodology; validation; visualization; writing – review and editing; software; formal analysis; data curation. **Stephanie L. Hardacre:** Conceptualization; project administration; supervision; resources; writing – review and editing; investigation. **Olivia Evans:** Conceptualization; investigation; funding acquisition; writing – original draft; writing – review and editing; project administration; supervision; resources. **Anika S. Quayle:** Conceptualization; investigation; writing – review and editing. **Mark Rubin:** Conceptualization; investigation; writing – review and editing; visualization; supervision. **Alysia Robertson:** Writing – original draft; writing – review and editing; methodology; formal analysis; software; data curation. **Tegan Cruwys:** Conceptualization; investigation; writing – review and editing; project administration; supervision; methodology. **Lillian Smyth:** Conceptualization; investigation; writing – review and editing; methodology; project administration; supervision.

## FUNDING INFORMATION

The work was supported by funding from the Australian Research Council (IN200100047). The funder had no role in the design of the study, data analysis or the decision to publish.

## CONFLICT OF INTEREST STATEMENT

The authors declare no conflict of interest.

## ETHICS STATEMENT

The authors assert that all procedures contributing to this work comply with the ethical standards of the relevant national and institutional committees on human experimentation and with the Helsinki Declaration of 1975, as revised in 2008. This study was approved by the Human Research Ethics Committee of the relevant university (protocol #2021/817).

## Supporting information


Data S1.

## Data Availability

The data that support the findings of this study are available on request from the corresponding author. The data are not publicly available due to privacy or ethical restrictions.

## Footnotes

^1^
Since both social support variables were positively skewed, a sensitivity analysis was run, in which a square root transformation was performed. This did not substantially change the results. As such, the results with the raw scores were reported.
