# Supplementary material for: Rethinking social media metrics: Daily interaction valence and well‐being in university students
Source: Br J Soc Psychol. 2026 Jul 3;65(3):e70109. doi: 10.1111/bjso.70109 (PMC13332309; doi:10.1111/bjso.70109)
Supplement: Supplementary file 1 — Data S1. [file BJSO-65-0-s001.docx]

**Supplementary Materials**

**R Code**

## uploading the files

dailywide <- read.csv("dailydiarywide.scales.csv")

View(dailywide)

baseline <- read.csv("baseline.scales.csv")

View(baseline)

ses <- read.csv("pre.standandized.ses.csv")

View(ses)

### code to switch the data from long to wide

names(dailywide)[1] <- "id"

install.packages("reshape2")

library(reshape2)

dailylong <- reshape(data=dailywide,

varying = 2:50,

timevar=c("day"),

idvar="id",

direction="long",

sep="_")

## merging the two files together, so we can have the baseline scores

# those that were done pre-survey with the daily diary data

# social class was taken in wave 1 tho

dailylongfull <- merge(dailylong, baseline, by = "id")

# the below was written because the original data file called baseline

# has social class as a z-score, while the ses data file has it as a raw score

dailylongfull <- merge(dailylong, ses, by = "id")

###################################################################

# need to recode the valence variable, to remove na/8

install.packages("dplyr")

library(dplyr)

dailylongfull$valence <- ifelse(dailylongfull$valence == 8,NA, dailylongfull$valence)

View(dailylongfull)

## mean centering (grand and individual center)

install.packages("dplyr")

library(dplyr)

install.packages("tidyverse")

library(tidyverse)

# key variables

dailylongfull <- dailylongfull |>

group_by(id) |>

mutate(

valence.cm = mean(valence, na.rm = TRUE),

valence.cwc = valence - valence.cm

) |>

ungroup()

dailylongfull <- dailylongfull |>

group_by(id) |>

mutate(

community.cm = mean(community, na.rm = TRUE),

community.cwc = community - community.cm

) |>

ungroup()

dailylongfull <- dailylongfull |>

group_by(id) |>

mutate(

SSprovided.cm = mean(SSprovided, na.rm = TRUE),

SSprovided.cwc = SSprovided - SSprovided.cm

) |>

ungroup()

dailylongfull <- dailylongfull |>

group_by(id) |>

mutate(

SSreceived.cm = mean(SSreceived, na.rm = TRUE),

SSreceived.cwc = SSreceived - SSreceived.cm

) |>

ungroup()

dailylongfull <- dailylongfull |>

group_by(id) |>

mutate(

MHI.cm = mean(MHI, na.rm = TRUE),

MHI.cwc = MHI - MHI.cm

) |>

ungroup()

##################################################

# because '8' for valence is not applicable, it is coded as missing in the main

#analysis but needs to be added back in, when running the missing data analysis

dailylongfull$valence[is.na(dailylongfull$valence)] <- 8

# missing data

library(dplyr)

dailylongfull %>%

summarise(across(

c(valence, MHI, community, SSprovided, SSreceived),

~ mean(is.na(.)) * 100

))

###########################################################################

library(lmerTest)

library(lme4)

###################################################

# computing the total effect pathway (c)

# c-path: Valence ➜ MHI (total effect)

model_c_mhi <- lmer(

MHI.cwc ~ valence.cwc + (1 | id),

data = dailylongfull,REML = FALSE

)

summary(model_c_mhi)

### social support provided

# a-path (moderated)

model_a_prov <- lmer(

SSprovided.cwc ~ valence.cwc * SES + (1 | id),

data = dailylongfull, REML = FALSE

)

summary(model_a_prov)

# no moderation

model_a_prov_simple <- lmer(

SSprovided.cwc ~ valence.cwc + (1 | id),

data = dailylongfull,REML = FALSE

)

summary(model_a_prov_simple)

# b-path and c′-path

model_b_prov <- lmer(

MHI.cwc ~ SSprovided.cwc + valence.cwc + (1 | id),

data = dailylongfull, REML = FALSE

)

summary(model_b_prov)

# c-path (total effect)

model_c_prov <- lmer(

MHI.cwc ~ valence.cwc + (1 | id),

data = dailylongfull, REML = FALSE

)

summary(model_c_prov)

a_prov <- fixef(model_a_prov_simple)["valence.cwc"]

b_prov <- fixef(model_b_prov)["SSprovided.cwc"]

indirect_effect_prov <- a_prov * b_prov

indirect_effect_prov

####################################################

### social support received

# a-path (moderated)

model_a_recv <- lmer(

SSreceived.cwc ~ valence.cwc * SES + (1 | id),

data = dailylongfull, REML = FALSE

)

summary(model_a_recv)

# no moderation

model_a_recv_simple <- lmer(

SSreceived.cwc ~ valence.cwc + (1 | id),

data = dailylongfull, REML = FALSE

)

summary(model_a_recv_simple)

# b-path and c′-path

model_b_recv <- lmer(

MHI.cwc ~ SSreceived.cwc + valence.cwc + (1 | id),

data = dailylongfull, REML = FALSE

)

summary(model_b_recv)

# c-path (total effect)

model_c_recv <- lmer(

MHI.cwc ~ valence.cwc + (1 | id),

data = dailylongfull, REML = FALSE

)

summary(model_c_recv)

a_recv <- fixef(model_a_recv_simple)["valence.cwc"]

b_recv <- fixef(model_b_recv)["SSreceived.cwc"]

indirect_effect_recv <- a_recv * b_recv

indirect_effect_recv

####################################################

### sense of community

# a-path (moderated)

model_a_comm <- lmer(

community.cwc ~ valence.cwc * SES + (1 | id),

data = dailylongfull, REML = FALSE

)

summary(model_a_comm)

# b-path and c′-path

model_b_comm <- lmer(

MHI.cwc ~ community.cwc + valence.cwc + (1 | id),

data = dailylongfull, REML = FALSE

)

summary(model_b_comm)

# c-path (total effect)

model_c_comm <- lmer(

MHI.cwc ~ valence.cwc + (1 | id),

data = dailylongfull, REML = FALSE

)

summary(model_c_comm)

# Visualise the fixed effects

library(ggplot2)

library(sjPlot)

mean(dailylongfull$SES, na.rm = TRUE)

sd(dailylongfull$SES, na.rm = TRUE)

plot_model(

model_a_comm,

type = "int",

terms = c("valence.cwc", "SES"),

mdrt.values = "meansd",

axis.title = c("Interaction Valence",

"Sense of Community"),

legend.title = "Social Class",

ci.lvl = 0.95

) +

theme_bw() +

scale_colour_grey(start = 0.2, end = 0.6) +

scale_colour_discrete(

labels = c("Low", "Mean", "High")

) +

labs(title = NULL) +

theme(

legend.position = "top",

text = element_text(size = 12),

panel.grid.major = element_blank(),

panel.grid.minor = element_blank(),

axis.title = element_text(face = "bold"),

legend.title = element_text(face = "bold"),

legend.key = element_blank()

)

# conditional indirect effects

a1_comm <- fixef(model_a_comm)["valence.cwc"]

a3_comm <- fixef(model_a_comm)["valence.cwc:SES"]

b_comm <- fixef(model_b_comm)["community.cwc"]

# SES values

SES_mean <- mean(dailylongfull$SES, na.rm = TRUE)

SES_sd <- sd(dailylongfull$SES, na.rm = TRUE)

SES_low <- SES_mean - SES_sd

SES_high <- SES_mean + SES_sd

# conditional a-paths

a_low_comm <- a1_comm + (a3_comm * SES_low)

a_mean_comm <- a1_comm + (a3_comm * SES_mean)

a_high_comm <- a1_comm + (a3_comm * SES_high)

# conditional indirect effects

indirect_low_comm <- a_low_comm * b_comm

indirect_mean_comm <- a_mean_comm * b_comm

indirect_high_comm <- a_high_comm * b_comm

indirect_low_comm

indirect_mean_comm

indirect_high_comm

# running community as a mediator, without social class as the moderator

#a- pathway

model_a_comm_simple <- lmer(

community.cwc ~ valence.cwc + (1 | id),

data = dailylongfull, REML = FALSE

)

summary(model_a_comm_simple)

# b-path and c′-path

model_b_comm1 <- lmer(

MHI.cwc ~ community.cwc + valence.cwc + (1 | id),

data = dailylongfull, REML = FALSE

)

summary(model_b_comm1)

# c-path (total effect)

model_c_comm1 <- lmer(

MHI.cwc ~ valence.cwc + (1 | id),

data = dailylongfull, REML = FALSE

)

summary(model_c_comm1)

a_comm <- fixef(model_a_comm_simple)["valence.cwc"]

b_comm <- fixef(model_b_comm1)["community.cwc"]

indirect_effect_comm <- a_comm * b_comm

indirect_effect_comm

#####################################################

# bootstrapping #

#####################################################

# social support provided

model_a_prov <- lm(

SSprovided.cwc ~ valence.cwc,

data = dailylongfull, REML = FALSE

)

model_b_prov <- lm(

MHI.cwc ~ SSprovided.cwc + valence.cwc,

data = dailylongfull, REML = FALSE

)

boot_fun_prov <- function(data, indices) {

d <- data[indices, ]

a_mod <- lm(SSprovided.cwc ~ valence.cwc, data = d)

b_mod <- lm(MHI.cwc ~ SSprovided.cwc + valence.cwc, data = d)

a <- coef(a_mod)["valence.cwc"]

b <- coef(b_mod)["SSprovided.cwc"]

ind <- a * b

return(ind)

}

# social support received

model_a_recv <- lm(

SSreceived.cwc ~ valence.cwc,

data = dailylongfull, REML = FALSE

)

model_b_recv <- lm(

MHI.cwc ~ SSreceived.cwc + valence.cwc,

data = dailylongfull, REML = FALSE

)

boot_fun_recv <- function(data, indices) {

d <- data[indices, ]

a_mod <- lm(SSreceived.cwc ~ valence.cwc, data = d)

b_mod <- lm(MHI.cwc ~ SSreceived.cwc + valence.cwc, data = d)

a <- coef(a_mod)["valence.cwc"]

b <- coef(b_mod)["SSreceived.cwc"]

ind <- a * b

return(ind)

}

# sense of community

model_a_comm_simple <- lm(

community.cwc ~ valence.cwc,

data = dailylongfull, REML = FALSE

)

model_b_comm1 <- lm(

MHI.cwc ~ community.cwc + valence.cwc,

data = dailylongfull, REML = FALSE

)

boot_fun_comm <- function(data, indices) {

d <- data[indices, ]

a_mod <- lm(community.cwc ~ valence.cwc, data = d)

b_mod <- lm(MHI.cwc ~ community.cwc + valence.cwc, data = d)

a <- coef(a_mod)["valence.cwc"]

b <- coef(b_mod)["community.cwc"]

ind <- a * b

return(ind)

}

library(boot)

boot_fun <- function(data, indices) {

d <- data[indices, ]

# a-path

a_mod <- lm(community.cwc ~ valence.cwc * SES, data = d)

# b-path

b_mod <- lm(MHI.cwc ~ community.cwc + valence.cwc, data = d)

# coefficients

a1 <- coef(a_mod)["valence.cwc"]

a3 <- coef(a_mod)["valence.cwc:SES"]

b <- coef(b_mod)["community.cwc"]

# SES values

SES_mean <- mean(d$SES, na.rm = TRUE)

SES_sd <- sd(d$SES, na.rm = TRUE)

SES_low <- SES_mean - SES_sd

SES_high <- SES_mean + SES_sd

# conditional indirects

ind_low <- (a1 + a3 * SES_low) * b

ind_mean <- (a1 + a3 * SES_mean) * b

ind_high <- (a1 + a3 * SES_high) * b

return(c(ind_low, ind_mean, ind_high))

}

set.seed(123)

boot_results <- boot(

data = dailylongfull,

statistic = boot_fun,

R = 5000

)

boot.ci(boot_results, type = "perc", index = 1) # low SES

boot.ci(boot_results, type = "perc", index = 2) # mean SES

boot.ci(boot_results, type = "perc", index = 3) # high SES

####

#running bootstrap for both social supports

library(boot)

set.seed(123)

boot_prov <- boot(dailylongfull, boot_fun_prov, R = 5000)

boot_recv <- boot(dailylongfull, boot_fun_recv, R = 5000)

boot_comm <- boot(dailylongfull, boot_fun_comm, R = 5000)

boot.ci(boot_prov, type = "perc")

boot.ci(boot_recv, type = "perc")

boot.ci(boot_comm, type = "perc")

###########################################################

# square root transformation for both social support variables

# social support provided

dailylongfull <- dailylongfull |>

mutate(

SSprovided_sqrt = sqrt(SSprovided)

)

dailylongfull <- dailylongfull |>

group_by(id) |>

mutate(

SSprovided_sqrt.cm = mean(SSprovided_sqrt, na.rm = TRUE),

SSprovided_sqrt.cwc = SSprovided_sqrt - SSprovided_sqrt.cm

) |>

ungroup()

# a-path (no moderation)

model_a_prov_simple1 <- lmer(

SSprovided_sqrt.cwc ~ valence.cwc + (1 | id),

data = dailylongfull,

REML = FALSE

)

summary(model_a_prov_simple1)

# b-path and c′-path

model_b_prov1 <- lmer(

MHI.cwc ~ SSprovided_sqrt.cwc + valence.cwc + (1 | id),

data = dailylongfull,

REML = FALSE

)

summary(model_b_prov1)

# c-path (total effect)

model_c_prov1 <- lmer(

MHI.cwc ~ valence.cwc + (1 | id),

data = dailylongfull,

REML = FALSE

)

summary(model_c_prov1)

a_prov1 <- fixef(model_a_prov_simple1)["valence.cwc"]

b_prov1 <- fixef(model_b_prov1)["SSprovided_sqrt.cwc"]

indirect_effect_prov1 <- a_prov1 * b_prov1

indirect_effect_prov1

####################################################

### social support received

dailylongfull <- dailylongfull |>

mutate(

SSreceived_sqrt = sqrt(SSreceived)

)

dailylongfull <- dailylongfull |>

group_by(id) |>

mutate(

SSreceived_sqrt.cm = mean(SSreceived_sqrt, na.rm = TRUE),

SSreceived_sqrt.cwc = SSreceived_sqrt - SSreceived_sqrt.cm

) |>

ungroup()

# a-path (no moderation)

model_a_recv_simple1 <- lmer(

SSreceived_sqrt.cwc ~ valence.cwc + (1 | id),

data = dailylongfull,

REML = FALSE

)

summary(model_a_recv_simple1)

# b-path and c′-path

model_b_recv1 <- lmer(

MHI.cwc ~ SSreceived_sqrt.cwc + valence.cwc + (1 | id),

data = dailylongfull,

REML = FALSE

)

summary(model_b_recv1)

# c-path (total effect)

model_c_recv1 <- lmer(

MHI.cwc ~ valence.cwc + (1 | id),

data = dailylongfull,

REML = FALSE

)

summary(model_c_recv1)

a_recv1 <- fixef(model_a_recv_simple1)["valence.cwc"]

b_recv1 <- fixef(model_b_recv1)["SSreceived_sqrt.cwc"]

indirect_effect_recv1 <- a_recv1 * b_recv1

indirect_effect_recv1

##### multiple imputation

install.packages("mice")

library(mice)

library(lme4)

library(lmerTest)

# select variables for imputation

imp_data <- dailylongfull[, c(

"id",

"day",

"SES",

"valence.cwc",

"SSprovided.cwc",

"SSreceived.cwc",

"community.cwc",

"MHI.cwc"

)]

# inspect missing data pattern

md.pattern(imp_data)

# initialise mice

ini <- mice(imp_data, maxit = 0)

pred <- ini$predictorMatrix

meth <- ini$method

# cluster variable

pred[, "id"] <- -2

# do not impute ID or day

meth["id"] <- ""

meth["day"] <- ""

# Level 2 variable (between-person)

meth["SES"] <- "2lonly.norm"

# Level 1 continuous variables

meth["valence.cwc"] <- "2l.pan"

meth["SSprovided.cwc"] <- "2l.pan"

meth["SSreceived.cwc"] <- "2l.pan"

meth["community.cwc"] <- "2l.pan"

meth["MHI.cwc"] <- "2l.pan"

# run imputation

imp <- mice(

imp_data,

m = 20,

method = meth,

predictorMatrix = pred,

maxit = 20,

seed = 1234

)

# check convergence

plot(imp)

######################################################

#social support provided

# double checking that there is no moderated result for provided

# Fit models to each imputed dataset

model_a_prov <- with(imp,

lmer(SSprovided.cwc ~ valence.cwc * SES + (1 | id), REML = FALSE)

)

# Pool the results

pool_a_prov <- pool(model_a_prov)

# View pooled summary

summary(pool_a_prov)

# continuing the model without moderation

# Fit models to each imputed dataset

model_a_prov_simple <- with(imp,

lmer(SSprovided.cwc ~ valence.cwc + (1 | id), REML = FALSE)

)

model_b_prov <- with(imp,

lmer(MHI.cwc ~ SSprovided.cwc + valence.cwc + (1 | id), REML = FALSE)

)

model_c_prov <- with(imp,

lmer(MHI.cwc ~ valence.cwc + (1 | id), REML = FALSE)

)

# Pool the results

pool_a <- pool(model_a_prov_simple)

pool_b <- pool(model_b_prov)

pool_c <- pool(model_c_prov)

# View pooled summaries

summary(pool_a)

summary(pool_b)

summary(pool_c)

#Extract pooled coefficients for indirect effect

pool_a_summary <- summary(pool_a)

pool_b_summary <- summary(pool_b)

a_prov <- pool_a_summary[pool_a_summary$term == "valence.cwc", "estimate"]

b_prov <- pool_b_summary[pool_b_summary$term == "SSprovided.cwc", "estimate"]

indirect_effect_prov <- a_prov * b_prov

indirect_effect_prov

#################################################################

library(boot)

# Function to calculate indirect effect for each bootstrap sample

indirect_effect_boot <- function(data, indices) {

boot_data <- data[indices, ]

model_a <- lmer(SSprovided.cwc ~ valence.cwc + (1 | id),

data = boot_data, REML = FALSE)

model_b <- lmer(MHI.cwc ~ SSprovided.cwc + valence.cwc + (1 | id),

data = boot_data, REML = FALSE)

a <- fixef(model_a)["valence.cwc"]

b <- fixef(model_b)["SSprovided.cwc"]

return(a * b)

}

# Bootstrap within each imputed dataset

boot_results <- list()

for (i in 1:imp$m) { # Loop through m imputed datasets

imp_complete <- complete(imp, i)

boot_results[[i]] <- boot(

data = imp_complete,

statistic = indirect_effect_boot,

R = 1000, # Number of bootstrap replicates

sim = "ordinary"

)

}

# Pool bootstrap results across imputations

# Extract bootstrap CIs from each imputation

boot_cis <- sapply(boot_results, function(x) {

boot.ci(x, type = "bca")$bca[4:5] # BCa CI bounds

})

# Average across imputations

pooled_ci_lower <- mean(boot_cis[1, ])

pooled_ci_upper <- mean(boot_cis[2, ])

pooled_ci_lower

pooled_ci_upper

######################################################################

#social support received

# double checking that there is no moderated result for received

# Fit models to each imputed dataset

model_a_rec <- with(imp,

lmer(SSreceived.cwc ~ valence.cwc * SES + (1 | id), REML = FALSE)

)

#Pool the results

pool_a_rec <- pool(model_a_rec)

# View pooled summary

summary(pool_a_rec)

# continuing the model without moderation

#Fit models to each imputed dataset

model_a_rec_simple <- with(imp,

lmer(SSreceived.cwc ~ valence.cwc + (1 | id), REML = FALSE)

)

model_b_rec <- with(imp,

lmer(MHI.cwc ~ SSreceived.cwc + valence.cwc + (1 | id), REML = FALSE)

)

model_c_rec <- with(imp,

lmer(MHI.cwc ~ valence.cwc + (1 | id), REML = FALSE)

)

#Pool the results

pool_a <- pool(model_a_rec_simple)

pool_b <- pool(model_b_rec)

pool_c <- pool(model_c_rec)

# View pooled summaries

summary(pool_a)

summary(pool_b)

summary(pool_c)

# Extract pooled coefficients for indirect effect

pool_a_summary <- summary(pool_a)

pool_b_summary <- summary(pool_b)

a_rec <- pool_a_summary[pool_a_summary$term == "valence.cwc", "estimate"]

b_rec <- pool_b_summary[pool_b_summary$term == "SSreceived.cwc", "estimate"]

indirect_effect_rec <- a_rec * b_rec

indirect_effect_rec

######################################################################

library(boot)

# Function to calculate indirect effect for each bootstrap sample

indirect_effect_boot <- function(data, indices) {

boot_data <- data[indices, ]

model_a <- lmer(SSreceived.cwc ~ valence.cwc + (1 | id),

data = boot_data, REML = FALSE)

model_b <- lmer(MHI.cwc ~ SSreceived.cwc + valence.cwc + (1 | id),

data = boot_data, REML = FALSE)

a <- fixef(model_a)["valence.cwc"]

b <- fixef(model_b)["SSreceived.cwc"]

return(a * b)

}

# Bootstrap within each imputed dataset

boot_results <- list()

for (i in 1:imp$m) { # Loop through m imputed datasets

imp_complete <- complete(imp, i)

boot_results[[i]] <- boot(

data = imp_complete,

statistic = indirect_effect_boot,

R = 1000, # Number of bootstrap replicates

sim = "ordinary"

)

}

# Pool bootstrap results across imputations

# Extract bootstrap CIs from each imputation

boot_cis <- sapply(boot_results, function(x) {

boot.ci(x, type = "bca")$bca[4:5] # BCa CI bounds

})

# Average across imputations

pooled_ci_lower <- mean(boot_cis[1, ])

pooled_ci_upper <- mean(boot_cis[2, ])

pooled_ci_lower

pooled_ci_upper

######################################################################

#sense of community

# checking for the moderator result

# Fit models to each imputed dataset

model_a_comm <- with(imp,

lmer(community.cwc ~ valence.cwc * SES + (1 | id), REML = FALSE)

)

model_b_comm <- with(imp,

lmer(MHI.cwc ~ community.cwc + valence.cwc + (1 | id), REML = FALSE)

)

model_c_comm <- with(imp,

lmer(MHI.cwc ~ valence.cwc + (1 | id), REML = FALSE)

)

#Pool the results

pool_a_comm <- pool(model_a_comm)

pool_b_comm <- pool(model_b_comm)

pool_c_comm <- pool(model_c_comm)

# View pooled summaries

summary(pool_a_comm)

summary(pool_b_comm)

summary(pool_c_comm)

######################################################################

#sense of community - without moderator

# Fit models to each imputed dataset

model_a_comm_simple <- with(imp,

lmer(community.cwc ~ valence.cwc + (1 | id), REML = FALSE)

)

model_b_comm1 <- with(imp,

lmer(MHI.cwc ~ community.cwc + valence.cwc + (1 | id), REML = FALSE)

)

model_c_comm1 <- with(imp,

lmer(MHI.cwc ~ valence.cwc + (1 | id), REML = FALSE)

)

# Pool the results

pool_a_comm_simple <- pool(model_a_comm_simple)

pool_b_comm1 <- pool(model_b_comm1)

pool_c_comm1 <- pool(model_c_comm1)

# View pooled summaries

summary(pool_a_comm_simple)

summary(pool_b_comm1)

summary(pool_c_comm1)

# Extract pooled coefficients for indirect effect

pool_a_comm_simple_summary <- summary(pool_a_comm_simple)

pool_b_comm1_summary <- summary(pool_b_comm1)

a_comm <- pool_a_comm_simple_summary[pool_a_comm_simple_summary$term == "valence.cwc", "estimate"]

b_comm <- pool_b_comm1_summary[pool_b_comm1_summary$term == "community.cwc", "estimate"]

indirect_effect_comm <- a_comm * b_comm

indirect_effect_comm

######################################################################

library(boot)

# Function to calculate indirect effect for each bootstrap sample

indirect_effect_boot_comm <- function(data, indices) {

boot_data <- data[indices, ]

model_a <- lmer(community.cwc ~ valence.cwc * SES + (1 | id),

data = boot_data, REML = FALSE)

model_b <- lmer(MHI.cwc ~ community.cwc + valence.cwc + (1 | id),

data = boot_data, REML = FALSE)

a <- fixef(model_a)["valence.cwc"]

b <- fixef(model_b)["community.cwc"]

return(a * b)

}

# Bootstrap within each imputed dataset

boot_results_comm <- list()

for (i in 1:imp$m) { # Loop through m imputed datasets

imp_complete <- complete(imp, i)

boot_results_comm[[i]] <- boot(

data = imp_complete,

statistic = indirect_effect_boot_comm,

R = 1000, # Number of bootstrap replicates

sim = "ordinary"

)

}

# Pool bootstrap results across imputations

boot_cis_comm <- sapply(boot_results_comm, function(x) {

boot.ci(x, type = "bca")$bca[4:5] # BCa CI bounds

})

# Average across imputations

pooled_ci_lower_comm <- mean(boot_cis_comm[1, ])

pooled_ci_upper_comm <- mean(boot_cis_comm[2, ])

pooled_ci_lower_comm

pooled_ci_upper_comm
